# Practitioner perspectives on extended difficulties and optimal support strategies following psychedelic experiences: a qualitative analysis

**DOI:** 10.1186/s12954-025-01347-0

**Published:** 2025-12-09

**Authors:** E. K. Argyri, J. Krecké, O. C. Robinson, J. Evans, M. Skragge, C. J. A. Morgan

**Affiliations:** 1 University of Exeter, Exeter, UK; 2 University of Greenwich, London, UK; 3 Challenging Psychedelic Experiences Project, London, UK; 4 Karlstad University, Karlstad, Sweden

**Keywords:** Psychedelics, Harm reduction, Integration, Post-psychedelic distress, Existential crisis, Trauma-informed care, Psychedelic-assisted therapy

## Abstract

**Background:**

As the use of psychedelics increases in both therapeutic and non-clinical settings, reports of extended post-experience difficulties have also emerged. While many individuals integrate their experiences effectively, others face persistent challenges. Despite growing recognition of these issues, there is limited research on best practices for mitigating and addressing prolonged post-psychedelic difficulties. This study explores expert perspectives on the nature of these challenges and optimal support strategies.

**Methods:**

A qualitative survey study was conducted with 28 professionals who support individuals navigating post-psychedelic distress, including psychiatrists, psychotherapists, psychedelic integration coaches, and retreat facilitators. Structured Tabular Thematic Analysis (ST-TA) was applied to identify high-consensus themes related to extended difficulties and effective integration practices.

**Results:**

Practitioners consistently reported six key post-psychedelic difficulties: (1) existential struggle and ontological shock, (2) anxiety and panic, (3) self-perception issues, (4) dissociative symptoms, (5) resurfacing of repressed material and trauma, and (6) disappointment due to unmet expectations. The most frequently recommended support strategies included (1) individual psychotherapy, particularly trauma-informed approaches, (2) grounding and mindfulness techniques, (3) peer and community support, (4) meaning-making and narrative reconstruction, and (5) in some cases, short-term psychiatric medication. While psychiatrists emphasized medical stabilization and symptom management, psychotherapists and integration coaches focused on existential meaning-making, emotional processing, and community-based support.

**Conclusions:**

Our findings highlight the need for trauma-informed, cross-disciplinary approaches to psychedelic integration. Ensuring access to ethical, evidence-based support—both clinical and community-based—is important for further developing harm reduction strategies as psychedelic use expands in the western world. Future research should explore culturally diverse integration practices and inform therapeutic protocols for mitigating post-psychedelic distress.

**Supplementary Information:**

The online version contains supplementary material available at 10.1186/s12954-025-01347-0.

## Background

Over the past three decades there has been a resurgence of research into the potential therapeutic benefits of psychedelic substances [1] with classical psychedelic compounds such as 5-MeO-DMT, DMT, LSD, mescaline and psilocybin, as well as ibogaine, ayahuasca, MDMA and ketamine, being investigated in clinical trials. Despite promising therapeutic outcomes across a range of psychedelics in clinical trials [2], there are risks associated with psychedelic use that remain only partly understood. The current study aims to explore conceptions of these risks, potential harms, and ways of supporting individuals who may be experiencing them, via various practitioners who work to support individuals during the integration or healing process after psychedelic experiences.

Psychedelic substances can induce intense subjective experiences characterized by altered sensory perceptions, heightened emotional sensitivity, and changes in thought patterns [3]. Such experiences can lead to lasting changes in personality, behavior, worldview and metaphysical beliefs [4–7] that in many cases are regarded as beneficial. However, these experiences can also be disruptive and challenging, particularly when adequate support is lacking [8–10]. Acute phases may involve overwhelming fear, paranoia, or confusion [8, 11, 12], and without appropriate support, these challenges may contribute to persistent negative psychological outcomes [8, 13, 14].

Surveys of naturalistic use highlight these risks: in one study of 770 Norwegian psychedelic users, 25% reported mild, transient adverse effects, while 4.2% reported lasting difficulties for over a year, and 2.9% reported flashbacks persisting for over a year [15]. In a survey of lifetime psychedelic users, 8.9% of 613 participants reported functional impairment lasting longer than a day as a result of such experiences [16]. In our team’s study focusing on individuals who had experienced extended difficulties after psychedelic use, a third of participants struggled for over a year and a sixth for more than three years [13].

As the use of psychedelics continues to rise in both recreational and therapeutic contexts, there is a growing need for structured support environments and evidence-based strategies to mitigate potential challenges. Two key approaches have emerged: harm reduction and integration [17]. Harm reduction focuses on minimizing immediate risks, offering education and real-time support during psychedelic experiences (e.g., festival harm reduction services, psychedelic helplines, therapeutic container) [18–20]. Psychedelic preparation, which includes psychoeducation, intention setting, and risk assessment, is a crucial component of harm reduction, as it can help mitigate adverse reactions and improve outcomes [21]. Integration, by contrast, is a post-experience process aimed at helping individuals make sense of their experiences, resolve distressing effects, and translate insights into lasting psychological growth [22]. While integration is widely acknowledged as crucial, current models vary significantly, spanning indigenous traditions, holistic practices, and psychotherapeutic frameworks [23]. Despite this diversity, there is no clear consensus on best practices, and psychedelic integration remains poorly standardized, with limited empirical research guiding its implementation [17, 24, 25].

While some research has sought to clarify best practices for integration, findings remain fragmented across different approaches. Clinical perspectives highlight structured therapeutic methods: Earleywine et al. [26] interviewed 30 clinical integration therapists, identifying key themes such as meaning-making, behavior change and supporting individuals in achieving a sense of wholeness. Expanding on this, Greń and colleagues [27] proposed a two-stage framework, emphasizing both pre-experience preparation (risk screening, psychoeducation, harm reduction) and post-experience integration (grounding techniques, emotional stabilization, normalization and structured meaning-making). In contrast, research on non-clinical integration practices reveals a more self-directed approach. Robinson and colleagues [10] found that recreational users rely on informal strategies such as cognitive reframing, spiritual practices, and peer support, with many reporting that simply being heard and validated was among the most helpful factors. Similarly, Wood, McAlpine and Kamboj [28] found preliminary evidence that acceptance, cognitive reappraisal and social support are strongly correlated with emotional breakthrough, which in turn has been suggested to be an important mediator for long-term psychological growth [29].

Despite three decades of renewed interest in psychedelic research, there remains a limited understanding of the adverse effects of psychedelic use and the most effective methods for preventing and alleviating prolonged difficulties [30]. Meanwhile, a diverse psychedelic ecosystem has burgeoned, comprising both overground and underground therapeutic practice, growing numbers of ‘recreational’ users and ceremonial or spiritual practices both in the global north and south. Much of the research in the ‘psychedelic renaissance’ has focused on clinical trials, which, while valuable, take place in controlled environments that do not reflect the real-world contexts in which psychedelics are used [31]. Recognizing this gap, O’Donnell and colleagues [32] have called for greater inclusion of practitioner perspectives in psychedelic research to enhance safety, therapeutic efficacy, and education. As demand for integration support continues to grow, further empirical work is needed to establish effective, evidence-based strategies for preventing and addressing post-psychedelic challenges.

## The current study

Our aim with the present study was to better understand post-psychedelic distress and effective support from the perspective of professionals who work in the field of supporting psychedelic users. The study explored expert opinions on the nature of post-psychedelic difficulties and the best practices for supporting individuals who have challenging experiences following the use of psychedelic substances. Given that expert opinions will vary depending on professions and backgrounds, we aimed to gather a diverse range of perspectives from psychiatrists, psychotherapists/psychologists, integration support coaches, and psychedelic facilitators. We sought out difficulties and support strategies that showed a high degree of consensus. We defined high consensus as being mentioned by at least three of the four participant groups (psychiatrist, psychotherapist, integration coach and psychedelic retreat facilitator), and being amongst the most frequently mentioned across the sample as a whole (see Data Analysis section for more detail). The two research questions that framed the study were as follows:

Research question 1: Which reported extended difficulties show a high degree of consensus across psychiatrics, psychotherapists, psychedelic integration coaches and retreat guides/facilitators, and how are these described?

Research question 2: What kinds of support and coping strategies for effectively addressing extended difficulties show a high degree of consensus across psychiatrics, psychotherapists, psychedelic integration coaches and retreat guides/facilitators, and how are these described?

## Method

### Participants and sample

The research team sought and contacted professionals across the four categories of psychiatrist, psychotherapist, integration coach and psychedelic retreat facilitator, who work with individuals who have experienced post-psychedelic difficulties. Potential participants were contacted via email and invited to take part in the study. Informed consent was obtained prior to survey administration. There were no financial incentives for participation. Inclusion criteria were that participants were over 18, fluent English speakers and that all participants were required to have worked with cases of post-psychedelic difficulties.

We used a multi-process recruitment strategy via contact networks, community outreach and snowball sampling, to contact individuals in psychotherapy, coaching, psychiatry and retreat guides who had experience of supporting people with post-psychedelic difficulties. 95% responded and participated. The process took four months and was terminated when we reached the a priori target for number and spread within the available resource envelope, also as per sampling guidance from Robinson [33].

The sample comprised 28 participants (*M* age = 44.96 years; range 30 – 75 years), evenly distributed across four types of practitioner groups (*n* = 7 each): psychiatrists, psychotherapists/psychologists, integration support coaches, and psychedelic retreat facilitators. On average, participants had worked in an area that interfaces with psychedelics for *M* = 9.73 years. Demographic frequencies of the sample are shown in Table 1.

**Table 1:** Frequencies and percentages of demographic categories within sample

		Frequency	Percentage of total sample (%)
Gender	Male	14	50
	Female	14	50
Age	30–39 years	5	18
	40–49 years	17	60
	50–59 years	5	18
	60 + years	1	4
Nationality	USA	8	29
	European (excl. UK)	6	21
	UK	5	18
	Canada	2	7
	Other/Mixed	7	25
Ethnicity	White	23	82
	Mixed	4	14
	Asian	1	4
Years worked with psychedelics	1–5 years	7	25
	6–10 years	12	43
	11–15 years	5	18
	15 + years	4	14

### Data collection

The study gained ethical approval from the University of Greenwich Research Ethics Board prior to the commencement of data collection (application ref: 23.3.5.11). The online survey platform Qualtrics was used to collect data. The two open-ended questions from the survey that are analysed in the current report are:

1. When clients or patients present with difficulties *after* using psychedelics, please describe the three difficulties you most commonly see? Please write a brief paragraph describing each one.
2. Please describe what you consider to be the best approaches or techniques for supporting individuals who present with the difficulties you previously described. You could write a brief paragraph for each type of difficulty previously mentioned, in terms of what you feel helps alleviate these different difficulties.

Other topics and associated questions were included in the survey and will be reported elsewhere. Participants were shown a debrief form at the end of the questionnaire, which provided information about support organisations and information websites that are orientated towards supporting individuals who have experienced difficulties with psychedelics and psychedelic integration.

### Qualitative analysis

Analysis of the data was conducted using Structured Tabular Thematic Analysis (ST-TA) [34]. This form of thematic analysis is specifically designed to analyse brief texts, such as social media comments or answers elicited by open-ended questions in questionnaires. It is epistemologically grounded in critical realism and thus operates on the premise that multiple interpretations of a phenomenon are inevitable—but that such interpretations can be evaluated for their relative veracity, based on evidence gleaned from a reality that is ontologically primary to human interpretations. ST-TA uses spreadsheet software to organize the data and thematising. The method has been used in over a hundred studies across the social sciences. It can be conducted using inductive, deductive or hybrid inductive-deductive approaches. Given the prior self-report studies with psychedelic users run by our research team that provided a basis for deductive coding, along with evolving and relatively new status of literature on post-psychedelic difficulties that provides a rationale for inductive coding, we chose to conduct an inductive- deductive hybrid analysis for the current study. The phases for inductive-deductive analysis in ST-TA are as follows: 1. Select the a priori Themes, 2. Deep Immersion in the Data; 3. Generating Revised Codes and Themes; 4. Tabulating Themes Against Data Segments; 5. Checking Inter-analyst Agreement; 6. Exploring Theme Frequencies, 6. Developing Thematic Maps and Tables, and 7. Producing the Report [34].

With regards to checking and developing agreement between analysts, this is part of ensuring that themes are transparently and cogently described and labelled, and of supporting eventual conclusions as consensual and not based on idiosyncratic interpretations pertaining to an individual researcher. ST-TA employs the agreement-reaching process to ensure that themes are sufficiently cogent and transparent for multiple analysts to code independently. The aim is to reach 80% agreement [34].

Two analysts (EKA, JK) split the dataset and reviewed responses to one question each. They took a hybrid approach, initially deductively coding against a set of themes from prior related publications [10, 13]. After initial coding they created themes to capture responses that did not clearly adhere to previous theme descriptors. Responses were coded independently initially, followed by a dialogical process of expounding the identified themes for each set of questions. Afterwards the analysts coded the responses to each other’s initially allocated question, blindly to the other’s prior coding. Agreement over 80% was reached for 45 out of 50 difficulties themes and for all of support themes in the first round of analysis. Remaining disagreements on the 5 difficulty themes were resolved through a discursive process involving OR, and themes were finalised accordingly [34].

*Research team and reflexivity:* The Challenging Psychedelic Experiences Project aims to contribute towards building a safer culture by studying and communicating psychedelic-induced difficulties and what helps overcome them. The multidisciplinary research team involved in this study was composed of six authors based in the UK, Costa Rica, and Sweden, with diverse nationalities and linguistic backgrounds. Three of the six researchers were native English speakers, and four resided in a country different from their country of birth. The team comprised three female and three male researchers. Reflecting on their positionality, four researchers reported having experienced extended difficulties following psychedelic use, while the remaining two had not. Five team members had experience supporting individuals through psychedelic-related challenges, either professionally (e.g., as clinical psychologists or integration facilitators) or in personal and peer-support capacities. This diversity of perspectives informed both the design and interpretive lens of the study.

*Selecting high consensus themes*: In order to determine which themes showed a high degree of consensus, we set an a priori requirement for a theme to be mentioned by at least three out of the four practitioner subsamples and to show a frequency that was 1 standard deviation or higher above the mean frequency (a frequency of 7 or above). The results section focuses on describing these themes. Other themes are presented in Tables 2 and 3.

## Results

In order to explore the high-consensus themes in detail, in line with our research questions, the narrative account of themes below focuses only on themes that reached our criteria for being high-consensus, with some additional example quotes to help illustrate them where beneficial. All themes that were elicited through the analysis process are shown in Tables 1 and 2 below, along with theme frequencies for each sub-sample (psychiatrists = PS, psychotherapists = PT, integration coaches = IC and retreat facilitators = RF) and for the sample as a whole. The tables thus evidence which themes were mentioned by which groups and to what extent. The themes are ordered from highest frequency of mention to lowest in the tables.

**Table 2:**
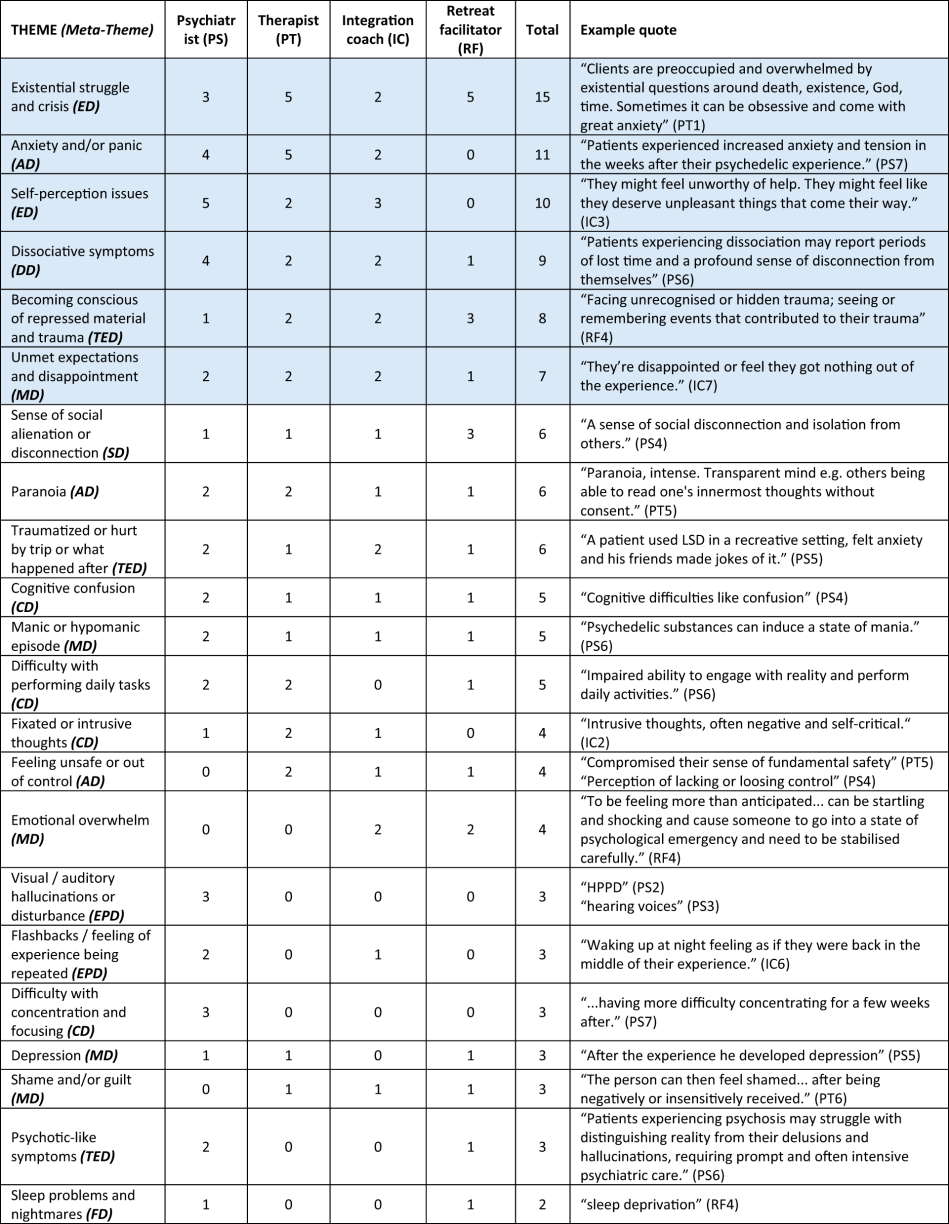

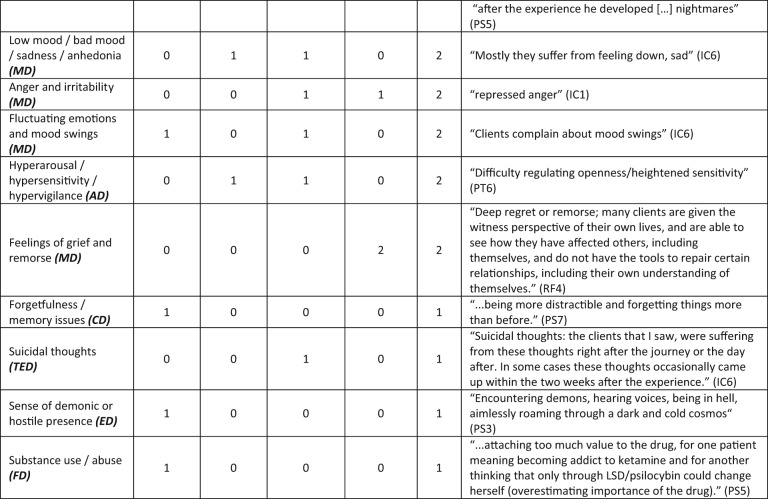
Difficulty themes, frequencies and example quotes, ordered from most prevalent in the sample to least prevalent

Themes that meet the criteria for high consensus are shaded blue

Abbreviations for nine difficulties meta-themes are included in brackets after theme names. Theme abbreviations: AD , anxiety difficulties; CD, cognitive difficulties; DD , dissociation difficulties; ED , existential difficulties; EPD,  reexperiencing and perceptual difficulties; FD,  functional difficulties; MD,  mood difficulties; SD,  social difficulties; TED , trauma and destabilisation difficulties

Participant identifier abbreviations: IC , integration coach; PS,  psychiatrist; PT,  psychotherapist; RF, retreat facilitator

### High consensus difficulty themes

**Table 3:**
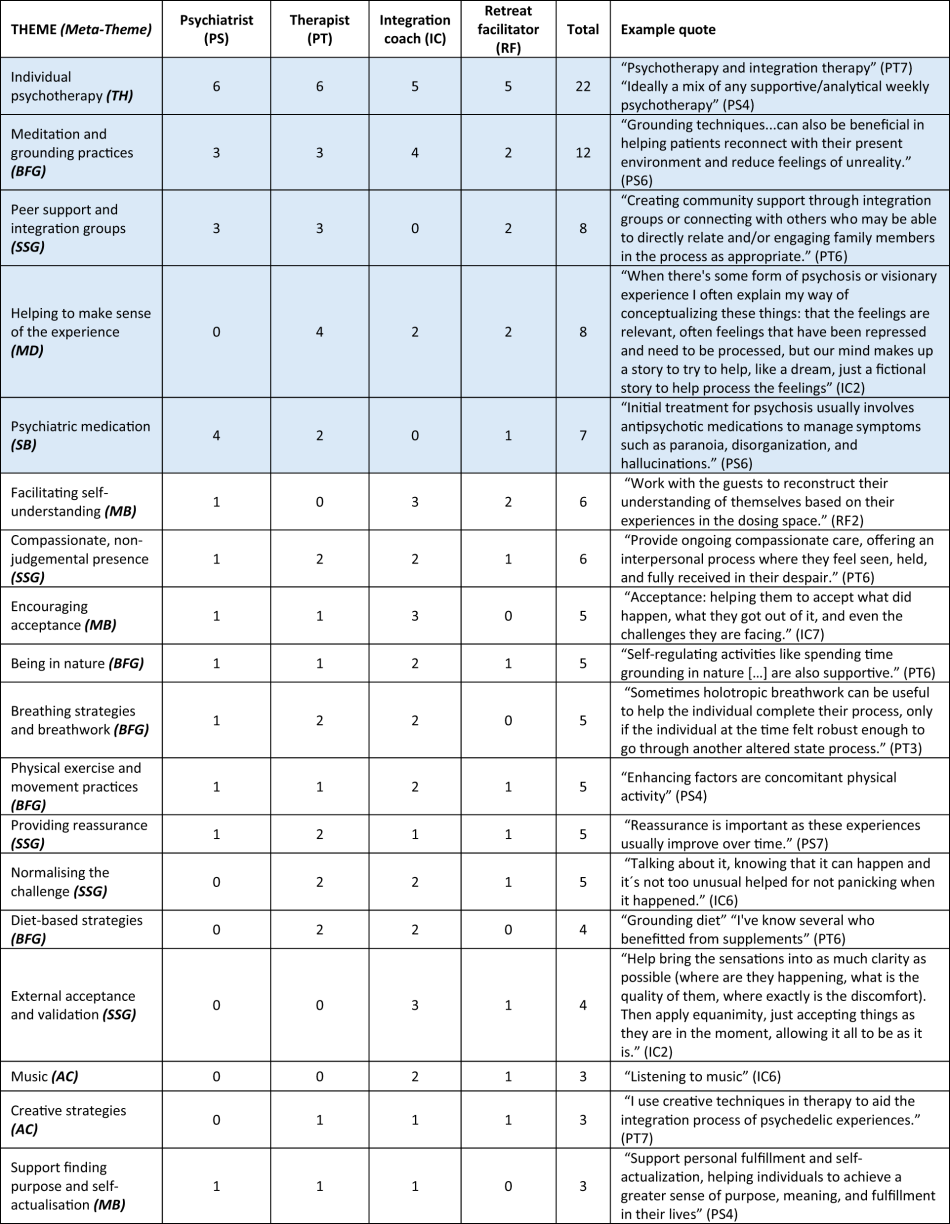

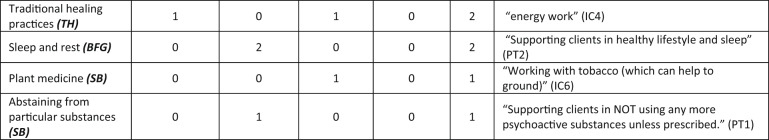
Support themes, frequencies and example quotes, ordered from most prevalent in the sample to least prevalent

Themes that meet the criteria for high consensus are shaded blue

Abbreviations for six support meta-themes are included in brackets after theme names. Theme abbreviations: AC , art and creative practices; BFG ,  body-focussed and grounding practices; MB , meaning-based practices; SB,  substance-based practices; SSG,  social support and guidance; TH , therapy and healing practices

Participant identifier abbreviations: IC,  integration coach; PS,  psychiatrist; PT,  psychotherapist; RF , retreat facilitator

Existential Struggle and Crisis
  *Existential Struggle and Crisis* was the most prevalent difficulty theme reported. It captured the discomfort, disorientation and distress associated with confronting existential matters of life’s meaning and the nature of reality after a psychedelic experience. Participants referred to how some of their clients ruminatively searched for answers to questions about death, existence, God, and time after their psychedelic experience, and encountering the anxiogenic sense that such sense-making may be impossible. One retreat facilitator described this as trying to grasp and compartmentalise what is ultimately unknowable or indivisible, which in turn leads to confusion and perseveration. A subtheme in existential struggle and crisis is *ontological shock*, where the nature of reality itself is thrown into question. Descriptions of ontological shock include irresolvable contradictions between transpersonal experiences and a materialistic paradigm, particularly in those with a rigid materialist worldview before the experience:
  *“Ontological shock: This can be when people have major transpersonal experiences without the necessary transpersonal or metaphysical frameworks to help them understand, find meaning in, and integrate their experiences.”* (RF3)
  *“…they're faced with the reality that what they thought ""reality"" was, was definitely wrong.”* (RF2)
  One participant mentioned that this is most common with DMT and ayahuasca:
  *“Some form of ontological shock - especially with DMT And Ayahuasca retreats. Individuals lack awareness of what to expect and have no frame of reference for their experiences. Clash of cosmologies - western mindset with shamanic cosmology.”* (PT3)
2. *Anxiety and/or panic*
  Descriptions of anxiety and/or panic were the second most common kind of difficulty. These include feelings of dread, of tension, of anxiety and panic attacks stemming from a profound sense of loss of control (e.g. loss of control of one’s own thoughts), and/or a loss of sense of self. One participant suggested that the anxiety was a reaction to an overwhelmed nervous system, which, in turn, they considered a function of trauma. Other participants referred to a fear of particular objects or a generalised sense of anxiety. Timescales were mentioned by participants that point to the fact that anxiety can last for days, weeks or months after the psychedelic experience. Two psychiatrists mentioned that the anxiety was an exacerbation or magnification of existing anxiety problems. Anxiety was also mentioned as linked to a hypomanic, agitated state after the experience.
Self-perception issues
  A further high consensus theme was *self-perception issues*. This relates to a range of distressing or dysfunction-inducing difficulties that pertain to the sense of self, other than dissociation. Self-perception issues related to either a sense of lacking a sense of “solidity of their own identity”, “doubting oneself” and “feeling unworthy”, or in contrast, ego inflation. Multiple mentions of ego inflation, grandiosity or ego aggrandisement were linked to manic or hypomanic behaviour. One participant described a client who was convinced of “being the new messiah that has to save the world” (PS5).
Dissociative Symptoms
  The *Dissociative Symptoms* theme pertains to depersonalisation and derealisation, and of related descriptions of psychological fragmentation and splitting. These experiences all relate a decoupling of the experiencing self—from the body, from psychic content such as thoughts or feelings, or from what they previously understood to be reality (derealisation). With this can come a disconnection from time or even from feeling alive. This was described as going on for months in some cases. Some example phrases used to capture this are as follows:
  “*Patients experiencing dissociation may report periods of lost time and a profound sense of disconnection from themselves, feeling as though the world around them is not real.”* (PS6)
  *“Clients have an unpleasant and lingering sense that things no longer feel real, or they themselves are no longer real.”* (PT1)
  *“Depersonalisation/Derealisation- feeling outside of one's body/self, feeling like things around them are not 'real' or difficult deciphering what is real and what is not”* (PT2)
Becoming conscious of repressed material
  Eight participants described the challenge that clients face when they feel that information has been brought into awareness that was previously repressed or hidden behind other defences. This can lead to a ‘flooding’ of trauma material into consciousness that a client then finds very difficult to contain. For some clients, the psychedelic experience was reported as generating highly anxiogenic questions about whether they were abused as a child. The possibility that these memories were paramnesia was also raised. This theme not only captures the emergence of memories into consciousness, but also the bringing of ‘shadow’ parts of the personality into conscious awareness, for example “murderous, violent or sexual fantasies and had previously assumed those belonged to other people, to bad people” (PT5). This return of repressed material was described as being particularly challenging “if people do not have the skills or inner resources to navigate very challenging material arising from the unconscious during the psychedelic experience” (RF3).
Unmet expectations and disappointment relating to experience and outcome
  Seven participants, distributed across all four sample groups, mentioned the matter of clients experiencing a deep sense of disappointment with the psychedelic experience not being curative or completely transformative, on the basis that they expected it to be a quick fix. One participant described how clients tend to have an expectation that the psychedelic experience will be a ‘silver bullet’. The difficulty with this is compounded when clients label the experience as not good, and then don’t engage with the integration phase or accept the need for work to be done after the experience itself.
  *“Expectations not met. People have great expectations about what psychedelics can do. They expect magic resolutions and are waiting to be “changed” by them without their own personal work.”* (PT4)
  *“Unmet expectations: Despite working to mitigate client's expectations of a "silver bullet solution" or full blown mystical experience" in the preparation phase, and despite psychoeducation around the integration phase being perhaps the most essential for the potential of transformative effects, clients sometimes express disappointment that the journey did not yield the impact they believed it would. Sometimes this leads them to not engage completely in the work of the integration phase that follows.”* (PT6)

### High consensus support themes

1. Individual psychotherapy
  The most common support theme (and the most common theme overall) was individual psychotherapy. This theme captures all explicit description of engaging in individual psychotherapy as a means of supporting someone with extended difficulties. Participants gave a range of good practice suggestions in terms of duration (weekly sessions until the client is stabilised), ethics (professional ethical training, for example on handling boundaries), that the practitioner should have strong knowledge of psychedelics, and should be trained in handling transference, intense emotions and splitting. Example quotes from participants are as follows:
  *“I think having licensed mental health professionals with experience working with altered states of consciousness work with these drugs for treatment of mental illness is a very good way to minimize these risks and manage them when they arise.”* (PS1)
  *“Working hand in hand with a therapist who can understand both realms the therapeutic and the psychedelic to be able to blend these two perspectives smoothly to provide a deep integration.”* (PT4)
  *“A strong therapeutic container, with the ability to find meaning in challenging experiences. A strong understanding on the part of the therapist on different metaphysical and psychological maps of consciousness.”* (RF3)
  A range of psychotherapeutic schools and techniques were coded as subthemes; Mindfulness-based CBT (2), Acceptance Commitment Therapy (2), hypnosis and trance (2), EMDR (4), trauma-informed therapy (7), Somatic therapies (including EFT), Psychoeducation techniques (5), Parts-work therapies on multiplicity of self (such as Internal Family Systems Therapy) (4), humanistic-holistic therapy (4), psychodynamic analytic therapies (1) and anger-reduction techniques (1).
2. Focusing on the present moment: meditation and grounding practices
  Meditation and grounding practices, both of which aim to bring attention into the present moment, were the second most common theme. They are a separate theme from individual therapy, as even though often mentioned as a component of individual therapy by some, they are mentioned in the context of self-care strategies, and group-based activities too. Grounding practices include the 5–4-3–2-1 technique, in which a person searches for five things they can see, four things they can touch, three things they can hear, two things they can smell and one thing they can taste. Participants mentioned that for participants who are dissociated or experiencing derealisation, returning to the embodied experiences of the present moment acts to help induce a sense of calm and reality. Example quotes include the following:
  *“mindfulness practices that encourage the client to move their awareness from their repetitive thoughts to sensations and observations. It helps to interrupt the pattern.”* (IC3)
  *“Grounding techniques and mindfulness practices can also be beneficial in helping patients reconnect with their present environment and reduce feelings of unreality.”* (PS6)
3. Community support: Peer/family support and integration groups
  Eight participants mentioned the healing role of community support via existing support networks, engaging family members if possible, and/or being part of integration groups of psychedelic users or sharing circles. The central component of this theme is countering a sense of disconnection with real connections, as described by this psychotherapist:
  *“Creating community support through integration groups or connecting with others who may be able to directly relate and/or engaging family members in the process as appropriate.”* (PT6)
4. Helping to make sense of the experience
  This theme overlaps with the Individual Psychotherapy theme, but was also mentioned as part of coaching initiatives, and as part of the encouragement to self-help. Examples of statements that were coded under this theme include suggestions of reading particular books to help make sense of the experience, encouragement to find connections between early experiences and current problems, or exploring alternative ways of making sense of visionary / psychotic experiences, such as making an equivalence with how dreams create fictional stories to help process emotions. The aim is to help participants to frame their experience in a way that allows for coherence and functional stability.
  “helping them to accept what did happen, what they got out of it, and even the challenges they are facing. This required helping them shift out of a victim approach. Authority over the narrative: helping them to create meaning and pull out messages that are meaningful to them.” (IC7)
  “work with the guests to reconstruct their understanding of themselves based on their experiences” (RF2)
5. Psychotropic medication
  Medications were mentioned as potentially helpful for individuals who are in distress or confused after a psychedelic experience. These include benzodiazepines, antipsychotic medication (if there is an underlying condition such as schizophrenia), mood stabilizers, atropine, anti-nausea medications, and natural substances with calming effects such as chamomile tea, L-Glutamine and magnolia extract.

## Discussion

This study contributes to the growing body of research on post-psychedelic difficulties and ways of supporting individuals who are going through difficulties, by adding insights from experienced practitioners to the previous research on this topic that has been based on data from psychedelic users.

### Themes pertaining to extended difficulties observed by practitioners

The most common difficulty theme was *Existential struggle and crisis*. The ontologically challenging content of a psychedelic trip can trigger or worsen existential challenges [8, 13, 14]. When psychedelic insights appear incompatible with an individual's prior worldview, they can destabilize ontological assumptions and trigger an existential disorientation in which core beliefs about reality, self, and existence are called into question [8, 35]. The practitioner perspectives in this study suggest that existential destabilization may be particularly pronounced in individuals with rigid ontological frameworks, such as strict materialist or religious worldviews..

Without appropriate preparation and frameworks for meaning-making, individuals may struggle with prolonged existential uncertainty, which in turn leads to anxiety and other functional disruptions [8]. A previous study found that existential struggle was the longest-lasting difficulty with a mean duration of 17 months [14]. Preparatory frameworks emphasizing philosophical flexibility, cognitive openness, and meaning-making strategies may help individuals navigate existential disruptions more adaptively. In addition, structured integration practices—such as existential therapy, guided philosophical inquiry, and narrative reconstruction—may support individuals in reframing their experiences in a coherent and constructive manner [36, 37].

The second most prevalent difficulty theme was *Anxiety and/or panic*. Practitioners observed that some individuals struggled with intrusive fears and heightened emotional sensitivity, often an exacerbation of prior anxiety. Psychiatrists noted that post-psychedelic anxiety could magnify pre-existing conditions or present as hypomanic agitation. This finding aligns with prior research showing that anxiety is one of the most common post-psychedelic difficulties, particularly when individuals enter altered states without adequate preparation or in challenging set and setting conditions [8, 10, 13, 38]. While many psychedelic experiences are characterized by transient anxiety that resolves as the experience unfolds [11], in some cases, the fear response persists beyond the acute phase, contributing to long-term distress [13]. It is possible that for individuals with pre-existing anxiety disorders or trauma histories the amplified emotional state overwhelms their regulatory capacity, leading to dysfunctional fear-processing [11, 13].

The third most prevalent difficulty theme was *Self-perception issues.* Practitioners conveyed the view that a destabilized sense of self brought on by psychedelic experiences can manifest as ego inflation, where individuals may either overidentify with their experience (grandiosity) or self-limiting doubt, the latter being a response to overwhelming uncertainty where a restricted but familiar self-concept may provide comfort. Prior research on ‘ego-dissolution’ in psychedelic experiences [39] has found that the blurring of self-boundaries is associated with a sense of oneness and often associated with therapeutic benefits, but may also lead to distress when individuals struggle to re-integrate their sense of self. Difficulties related to self-perception have been reported particularly in individuals with pre-existing vulnerabilities, such as history of trauma or psychiatric diagnoses and the interplay between set, setting and prior psychological state seems to be key in shaping whether ego-related disruptions lead to insight and positive transformation or prolonged distress [13].

The fourth and fifth most prevalent difficulty themes were *Dissociation* and *Becoming aware of repressed material.* Dissociation can emerge as an adaptive mechanism, that allows individuals to detach from the emotional intense states that are experienced as overwhelming [40, 41]. The link between highly stressful experiences and dissociation as a trauma response is well documented. Dissociation is sometimes conceptualised as an autonomic defence response to overwhelming stress, particularly within certain trauma frameworks [42]. However, this view is not universally accepted, and alternative perspectives highlight the complexity and heterogeneity of dissociative experiences [42]. While dissociation may help individuals cope in the short term, in the long term it is linked to poorer outcomes in people who have experienced stressful events, including hyper-vigilance and re-experiencing phenomena. It can complicate the integration process, leaving individuals emotionally fragmented and disconnected from their present experience, as reported by our participants.

The practitioners in our survey noted that individuals may encounter emotionally intense or destabilizing material, potentially triggering the resurfacing of unresolved traumas or a new traumatic experience [43–45]. This aligns with the review of Rougemont-Bücking and colleagues [46] which highlights that individuals may experience traumatic reactivations where destabilising and unresolved material arises during psychedelic sessions. They argue that if the patient and therapist successfully establish a sense of safety to navigate the resurfaced material, the resolution can be transformative and healing, while in absence of appropriate support the experience can trigger trauma and leading to worsened emotional dysregulation. In light of this, our findings emphasize the need for trauma-informed approaches (acknowledging the role that trauma may play in individuals lives) [47] and appropriate preparation [48].

The final high-consensus difficulty theme was *Unmet expectations and disappointment*. Previous research found that positive prior expectations predicted more positive psychedelic experiences [49, 50]. While some degree of positive priming can be beneficial—enhancing suggestibility and increasing the likelihood of a meaningful or therapeutic experience—overly inflated expectations may lead to disappointment, frustration, or disillusionment when an individual does not experience the profound transformation they anticipated. When psychedelic experiences fail to meet high expectations, individuals may disengage from integration work essential for long-term benefits.

As McAlpine and colleagues [51] argue, managing expectations through psychoeducation is essential to prepare individuals for the unpredictable nature of psychedelic experiences. Increasingly researchers in the field are highlighting the risks of psychedelic hype [8, 52–54] and calling for more balanced, rigorous studies and media reporting [55–57].

### Themes pertaining to practitioner perspectives on optimal support

The most commonly cited form of effective support was *Individual Psychotherapy*. Therapeutic approaches varied depending on practitioner background, and included CBT, ACT, EMDR, somatic therapy, psychodynamic therapy and more. The variety of techniques in our data reflects recent critiques of psychedelic research that highlight issues in the standardization of psychedelic-assisted psychotherapy [20]. This lack of rigor may contribute to an overemphasis on the pharmacological effects of psychedelic drugs while neglecting the role of psychotherapy in shaping outcomes, which further complicates informed decision-making for policy [58].

A pertinent contrast between the findings of this practitioner study and studies with psychedelic *users* about post-psychedelic difficulties, is the relative difference in how often therapy is mentioned across these two groups. Whereas therapy was the most commonly mentioned form of effective support by the practitioners, users mentioned community / peer support almost twice as frequently as therapy [10]. In another study that investigated which strategies that were effective for specific post-psychedelic difficulties, support from peers was seen as more effective than therapy for anxiety, and self-education as more helpful than therapy for existential struggle, derealisation/depersonalisation and paranoia [14]. These findings may reflect the fact that practitioners consider effective strategies through the lens of their role in the process as a therapist or guide and may be unaware of the proportion of users who employ more informal channels of support and information.

The second most commonly mentioned support theme was *Meditation and grounding practices*. Grounding techniques were widely recommended for managing dissociation, derealization and ontological shock. These practices help individuals reconnect with the present moment and physical experience, reducing emotional distress. Our previous work has shown that individuals experiencing post-psychedelic distress often report benefit from strategies that helped them reconnect with the present moment [10] and various grounding practices ranging from experiences with water, exercise, yoga to creative practices, singing and playing instruments [8], [59, 60].

The value of community-based integration was another high-consensus support theme. This confirms our previous findings that peer and family support are highly effective in managing post-psychedelic challenges when there’s a lack of professional resources, [10] and can offer continuity beyond retreat settings [61]. Community spaces provide opportunity for shared reflection, storytelling, and mutual validation, helping individuals find meaning, navigate difficult emotions and share coping strategies [8, 62], [63].

## Limitations and future directions

This study is limited to perspectives from practitioners in the Western world, whose training in Western medicine and academia may shape their approaches to psychedelic-related difficulties. Traditional and indigenous healing practices, such as Amazonian ayahuasca ceremonies, often integrate psychedelic use within communal and spiritual frameworks, which contrast with the individualistic and biomedical paradigms of Western psychiatry [64]. These differing epistemologies likely influence how extended difficulties are conceptualized, diagnosed, and treated [65]. To address this gap, we begun data collection for a separate study exploring indigenous practitioner perspectives.

Furthermore, with a sample of 28 participants, this study provides an initial exploration of perspectives from psychiatrists, psychotherapists, integration coaches, and retreat facilitators, identifying key areas of consensus in managing post-psychedelic distress across these diverse practitioners. We hope that our study will provide a basis for deductive work to examine the applicability of our themes in other samples and contexts. Larger, more diverse samples are needed to further validate these findings and capture potential divergences across practitioner groups. One direction for future research is to compare the practitioner groups we have identified here with a survey and larger sample to explore if there are systematic discrepancies in opinions across the groups. For example, our data suggest that psychiatrists focused on clinical diagnoses, pre-existing conditions and pharmacological interventions, while psychotherapists tended to emphasize meaning-making and integration. Integration coaches and retreat facilitators, in contrast, focused on self-regulation techniques and the importance of community support. These differences reflect both professional roles and the phases in which practitioners encounter clients. All agreed on the importance of psychotherapy. Additional variables to consider in further research with practitioners are the beliefs of the practitioner (their ontological frameworks) in relation to psychedelics (for example, do they believe ‘the medicine’ is an animate benevolent spirit who always knows what a client needs to experience) and the extent to which they consider themselves to be part of psychedelic culture, psychedelic spirituality or ‘the psychedelic movement’.

Additionally, our focus on practitioner perspectives regarding the phenomenology and support strategies for extended difficulties prioritizes the integration phase of psychedelic experiences. Future research should examine harm reduction approaches across different settings (including therapeutic, recreational, self-development use), exploring optimal practices during the preparatory and acute phases of the psychedelic experience.

Our study provides insights into practitioner perspectives of post-psychedelic extended difficulties and forms of support. Different difficulties may emerge and relate to different phases of the integration process and tailored support may be necessary. Future studies can explore the optimization of support based on the trajectory of the difficulties development.

Finally, while we focus on high consensus themes within our sample, our findings are to be interpreted as preliminary; the limitations of our sample mean that claims for a wider consensus in the field are pending further research. A large Delphi study that includes practitioners from different settings and cultures as well as both psychedelic therapy patients and recreational users’ perspectives is needed to help us identify consensus points for informing psychedelic harm reduction. Such a large consensus study may help towards identifying diagnostic criteria and recommending treatments for the most common post-psychedelic difficulties, which could then be communicated to the psychedelic industry and to psychiatric and psychotherapeutic practitioners.

## Conclusions: implications and suggestions for the field

Our findings emphasize the need for structured harm reduction and integration strategies in supporting individuals struggling with post-psychedelic difficulties. The challenges reported by practitioners—such as existential distress, anxiety, dissociation, resurfacing trauma, and self-perception issues—highlight key areas where improved intervention models and systemic support structures are necessary. Trauma-informed care may be especially important given that individuals with prior trauma may be at increased risk of destabilisation. Training psychedelic practitioners in trauma-informed modalities may help mitigate these risks. Evidence-based trauma-focused treatments such as EMDR may be appropriate in some cases. Other modalities commonly used in psychedelic integration—such as somatic approaches, Internal Family Systems (IFS), and mindfulness-based practices—may support meaning-making and emotional processing, though their empirical support remains limited and contested [66]. Further research is needed to evaluate these approaches in the context of psychedelic-related care.

Our study highlights the role of unrealistic expectations in contributing to post-experience disappointment, disengagement from integration work, and prolonged distress. The commercialization and mainstreaming of psychedelics have fuelled media narratives that frame these substances as transformative ‘magic pills’, often failing to acknowledge the risks and complexities involved. Harm reduction organizations, therapists, and researchers ought to work toward more realistic messaging that frames psychedelic experiences as tools for growth that require effortful integration, rather than guaranteed cures.

Community-based integration practices were widely emphasized by practitioners as a key factor in mitigating distress and fostering psychological resilience post-experience. Given that therapeutic support is often inaccessible due to cost or availability, peer-led and community-based models may provide scalable, accessible alternatives for individuals seeking integration support. In addition, cultural integration models found in indigenous and traditional psychedelic-using contexts could serve as valuable templates for fostering collective meaning-making and support structures.

## Supplementary Information

Below is the link to the electronic supplementary material.

Supplementary Material 1

## Data Availability

Data is provided within the manuscript and the supplementary information files.
